# Sequence determinants of innate immune activation by short interfering RNAs

**DOI:** 10.1186/1471-2172-10-40

**Published:** 2009-07-24

**Authors:** Amber Goodchild, Nicole Nopper, Andrew King, Tram Doan, Marcel Tanudji, Greg M Arndt, Michael Poidinger, Laurent P Rivory, Toby Passioura

**Affiliations:** 1 Johnson and Johnson Research Pty Ltd, Locked Bag 4555, Strawberry Hills NSW, Australia

## Abstract

**Background:**

Short interfering RNAs (siRNAs) have been shown to induce immune stimulation through a number of different receptors in a range of cell types. In primary cells, both TLR7 and TLR8 have been shown to recognise siRNAs however, despite the identification of a number of TLR7/8 stimulatory RNA motifs, the complete and definitive sequence determinants of TLR7 and TLR8 are yet to be elucidated.

**Results:**

A total of 207 siRNA sequences were screened for TLR7/8 stimulation in human PBMCs. There was a significant correlation between the U count of the U-rich strand and the immunostimulatory activity of the duplex. Using siRNAs specifically designed to analyse the effect of base substitutions and hybridisation of the two strands, we found that sequence motifs and the thermodynamic properties of the duplexes appeared to be the major determinants of siRNA immunogenicity and that the strength of the hybridisation interaction between the two strands correlated negatively with immunostimulatory activity.

**Conclusion:**

The data presented favour a model of TLR7/8 activation by siRNAs, in which the two strands are denatured in the endosome, and single-stranded, U-rich RNA species activate TLR7/8. These findings have relevance to the design of siRNAs, particularly for *in vivo *or clinical applications.

## Background

The mammalian innate immune system utilises a number of protein receptors for the identification of microbial molecules. Amongst these are several receptors specific for foreign ribonucleic acids (RNAs), including Toll-like receptor 3 (TLR3), TLR7, TLR8, RIG-I, MDA5 and PKR (reviewed in [1]). These receptors are specifically activated by different microbial RNA features, including double-strandedness (TLR3, RIG-I, MDA5, PKR) [2-4], extracellular or endosomal localization (TLR3, TLR7, TLR8) [5,6] and/or the presence of 5'-triphosphates (RIG-I, PKR) [7-9]. The engagement of these receptors by their cognate ligands induces the expression of anti-viral genes, including cytokines and type I interferons (IFNs).

Short interfering RNAs (siRNAs) are routinely used in laboratory settings, and hold promise for a range of therapeutic applications [10,11]. Although initially thought to bypass the innate immune system by virtue of their size [12,13], siRNAs have more recently been shown to induce immune stimulation in a variety of *in vivo*, and *in vitro *settings (reviewed in [14-16]). Synthetic siRNA duplexes have now been show to activate PKR in glioblastoma cells [17], RIG-I in glioblastoma cells and primary human monocytes [7,18], TLR3 in HEK293 cells and murine models [19,20], TLR7 in murine leukocytes and human plasmacytoid dendritic cells [21], and TLR8 in human monocytes [22]. In addition, a number of groups have demonstrated innate immune stimulation by siRNAs in human peripheral blood mononuclear cells (PBMCs) without defining the cell or receptor specificity [23-26].

Whilst the activation of RIG-I, PKR and TLR3 described above does occur with specific types of siRNA and in specific cellular contexts, it appears that the major innate immune response to standard 21 mer siRNAs (*i.e*. without 5'-triphosphates and with 3'-overhangs) is activation of TLR7 and/or TLR8 in leukocytes [21-26]. In isolated human leukocytes immune stimulation has been shown to be mediated by TLR7/8 activation since it is dependent upon endosomal maturation (excluding activation of cytosolic receptors such as RIG-I), and mediated by single-stranded RNA in a sequence-dependent fashion (excluding TLR3) [21,22,24,26]. Sequence-dependent activation of TLR7 by such siRNA has been demonstrated in primary human pDCs, murine leukocytes and in *in vivo *murine models [21]. Sequence-dependent activation of human monocytes through an endosomal pathway has also been reported [22], presumably through activation of TLR8 which is the primary sensor of single-stranded RNA in these cells [27]. Stimulation of human PBMCs with siRNA can lead to production of either IFNα or TNFα or both simultaneously, leading to the hypothesis that differences in the sequence specificity of TLR7 and TLR8 may cause a given immunostimulatory sequence to activate pDCs through TLR7 and/or monocytes through TLR8 [26].

TLR7 and TLR8 are both activated by single-stranded RNA. The precise sequence requirements for their activation have not yet been elucidated, however, it has been demonstrated that G and U rich sequences tend to stimulate TLR7 causing IFNα production from pDCs, and A and U rich sequences tend to stimulate TLR8 causing production of both IFNα and TNFα from monocytes [28-30]. It has not yet been determined whether these base preferences also affect the activation of TLR7/8 by siRNA (it should be noted that for an siRNA duplex of a given length, the total G+U content is identical irrespective of the sequence and is equal to the length of the siRNA). However, it has been shown that TLR7/8 immunostimulatory duplex siRNAs are less active than their component single strands, suggesting that activation of TLR7/8 by siRNAs is induced by the latter [31]. In support of this hypothesis, specific G and U rich TLR7 stimulatory motifs (GUCCUUCAA and UGUGU) have been identified in the single strand components of two siRNA duplexes [21,24].

Thus, it appears that the primary mechanism of innate immune stimulation by siRNAs is the activation of TLR7/8 by a single strand of the siRNA duplex (which may become denatured in the endosome, allowing release of the immunostimulatory strand). In the present study, we have investigated the sequence requirements for siRNA stimulation of TLR7/8 by screening 207 siRNA sequences for stimulation of human PBMCs. We found that sequence motifs and the thermodynamic properties of the duplexes appeared to be the major determinants of siRNA immunogenicity. In addition, we have identified a number of highly immunogenic RNA sequences.

## Results and Discussion

### Assay development

In order to determine the determinants of immunogenicity of siRNAs, we initially sought to develop a sensitive and relatively high-throughput assay for the detection of innate immune stimulation by short duplex RNAs. The logical choice of target cells for such an assay is primary human PBMCs, which have been shown to produce pro-inflammatory cytokines and IFN in response to siRNAs, through activation of TLR7 and/or TLR8 [21,22,24-26]. These primary cells have the additional advantage that, unlike TLR7 transfected immortalised cell lines, they appear to fully recapitulate *in vivo *TLR signalling [30]. We and others [32,33], had shown that the human hepatoma cell line Huh7, harbouring an HCV replicon that expresses a luciferase reporter, is a sensitive and easily assayed system for detection of IFN. Therefore, we chose to employ an assay system in which innate immunostimulation of PBMCs by siRNAs is detected using inhibition of HCV replication as a surrogate marker for IFN production. Such assay systems have been shown to be dependent on immune stimulation of the leukocytes (since treatment of Huh7 cells with a broad range of TLR agonists in the absence of leukocytes does not inhibit replication of the HCV replicon) and to be dependent upon secretion of IFNα from the activated leukocytes (as shown by neutralizing mAb experiments) [32,33].

**Figure 1:**
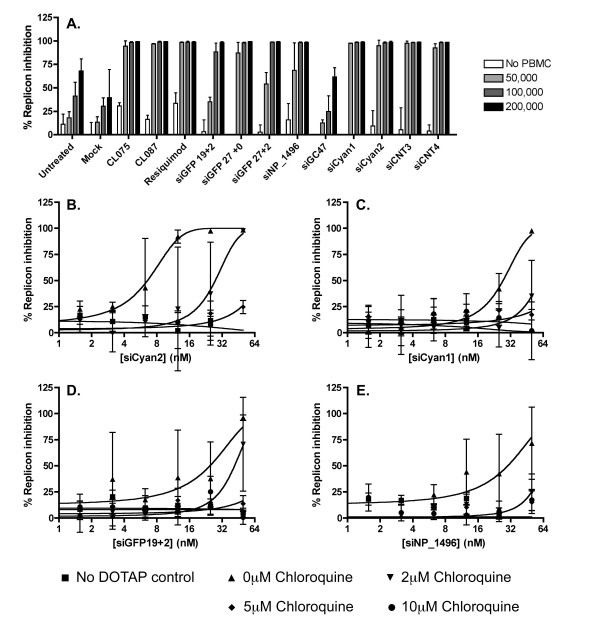
**Optimization of assay for detection of innate immune stimulation of PBMCs by siRNA**. Supernatant from PBMCs treated with DOTAP complexed siRNAs for 24 hours was applied to Huh7-Luc cells. Luciferase expression was assayed 24 hours after treatment with supernatant. Each point represents the mean of triplicate samples. Error bars indicate standard deviation. (**A**) The indicated densities (cells/100 μL) of PBMCs were stimulated with 40 nM indicated siRNAs. (**B**)-(**E**) Dose response curves performed using PBMCs at a density of 50,000/100 μL in the presence or absence of chloroquine at the indicated concentrations.

To determine the optimal density of PBMCs for such an assay, non-cryopreserved primary human PBMCs at densities of 5–20 × 10^4 ^cells/100 μl were treated with 50 nM (a comparable concentration to previous studies references 21–26) siRNAs complexed with DOTAP (which has been shown to be the most effective complexation agent for the detection of innate immune activity of siRNAs [26]). After 24 hours, the supernatant was transferred to Huh7-Luc cells, which were incubated for a further 24 hours to allow inhibition of HCV replication by IFN, before luciferase expression was assayed. A range of different siRNAs were tested. Of these, three (siGFP19+2, siGFP27+0 and siGFP 27+2) were variants of the same GFP targeting siRNA, which had been shown to induce cytokine and IFN induction in PBMCs [26] and differed only in length and the presence or absence of 3' 2 nucleotide overhangs; one (siNP_1496) was an Influenza A targeting siRNA which had been shown to induce off-target immune effects [25]; three (siGC47, siCNT3, siCNT4) were commercially obtained control siRNA without target sites in the human transcriptome; and two (siCyan1 and siCyan2) were designed to target a cyan fluorescent protein gene. In the absence of siRNA (both in untreated PBMCs and PBMCs treated with DOTAP alone) there was a cell density-dependent suppression of HCV replication (presumably due to constitutive IFN production from the PBMCs) with background HCV suppression of the order of 50% at a density of 20 × 10^4 ^PBMC/100 μL (Figure 1A). By contrast, background HCV inhibition was slight at a density of 5 × 10^4 ^PBMCs/100 μL. At this density, HCV inhibition was observed following treatment of the PBMCs with almost all of the siRNAs, with 4 siRNAs inducing 100% HCV inhibition (Figure 1A). Thus, 5 × 10^4 ^PBMCs/100 μl appeared to provide the necessary dynamic range for assaying innate immune stimulation by siRNAs, and this density was used for further experiments.

In order to demonstrate that the PBMC/Huh7-Luc assay system was capable of measuring differences in immunostimulatory activity between different siRNAs, we assessed the dose responses of 4 of the above mentioned siRNA: 2 strong immunostimulants (siCyan1 and siCyan2) and 2 moderate immunostimulants (siGFP19+2 and siNP_1496). Each of these duplexes induced a dose-dependent response from PBMC, with clear differences between the activity of each detectable in this assay (Figure 1B–E). This response was dependent upon endosomal maturation (and was therefore not mediated by cytosolic RNA receptors), since pre-treatment of the PBMCs with chloroquine inhibited IFNα production in a dose-dependent manner.

### Screening siRNA duplexes for TLR7/8 activation

**Figure 2:**
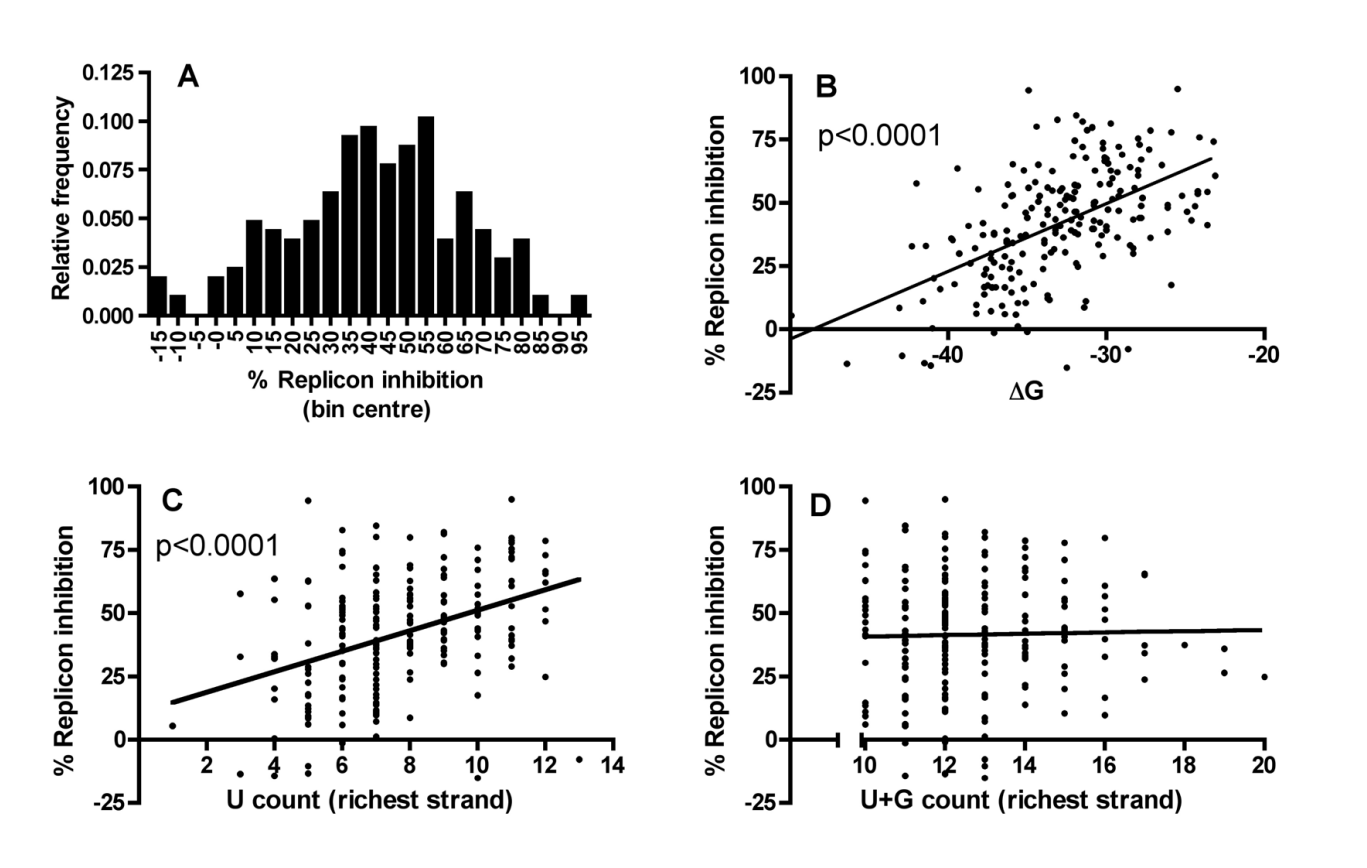
**Innate immune stimulation of PBMCs by 207 siRNAs**. Supernatant from PBMCs treated with 100 nM DOTAP complexed siRNAs for 24 hours was applied to Huh7-Luc cells. Luciferase expression was assayed 24 hours after treatment with supernatant. Each point represents the mean of triplicate samples. (**A**) Frequency histogram of replicon inhibition by all 207 siRNAs in 5% bins. (**B**) Correlation between replicon inhibition an d free energy of hybridisation (ΔG) for all 207 siRNAs. (**C**) Correlation between replicon inhibition and U content for the strand of the duplex with higher U content (or both strands for duplexes with equal U content in both strands). (**D**) Correlation between replicon inhibition and U+G content for the strand of the duplex with higher U+G content (or both strands for duplexes with equal U+G content in both strands).

Having established a working assay for TLR7/8 activation by siRNA, we sought to identify the sequence determinants of this activation. We performed a 4-point (1.56, 6.25, 25 and 100 nM) dose response curve for 207 siRNA duplexes (21 nucleotide strands with 2 base 3' overhangs – see Additional File 1) complexed with DOTAP. At a concentration of 100 nM, we observed a range of innate immune stimulation from undetectable up to the maximum quantifiable by the assay (Figure 2A). The immunostimulation observed was dependent upon endosomal maturation, since pre-treatment of the PBMCs with chloroquine abrogated replicon suppression for every siRNA duplex tested (data not shown). Since it seemed probable that denaturation of the strands was required for activation of TLR7/8, we compared the observed suppressive activity with the free energy of hybridisation of the duplex. We found a significant correlation (p < 0.0001) between this parameter and immunostimulatory activity (Figure 2B), suggesting that denaturation of the strands was indeed required for activation of TLR7/8.

Although apparently affected by characteristics of the duplex (such as the free energy of hybridisation as discussed above), immune activation of PBMCs by siRNAs generally appears to be mediated by a single U rich strand of the siRNA [21,31]. We compared the U content of both strands in each of the 207 duplexes, and selected the strand with the higher U content as being the probable immunostimulatory strand. Consistent with this model, there was a significant correlation between the U count of the U-rich strand and the immunostimulatory activity of the duplex (Figure 2C). G content has also been postulated to promote activation of TLR7/8 [21], however, we saw no correlation between combined U+G content and activity (Figure 2D).

**Figure 3:**
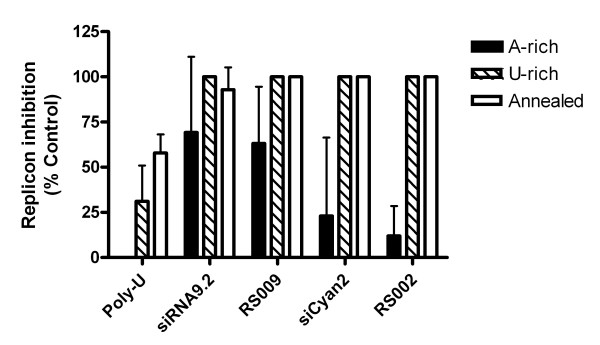
**Innate immune stimulation of PBMCs by single-stranded and annealed siRNAs**. Supernatant from PBMCs treated with 100 nM DOTAP complexed RNAs for 24 hours was applied to Huh7-Luc cells. Luciferase expression was assayed 24 hours after treatment with supernatant. Each point represents the mean of triplicate samples.

The above analysis made the assumption that the more U rich strand of each duplex was responsible for the majority of the immunostimulatory activity observed for that duplex. To assess the validity of this assumption, we investigated the immunostimulatory activity of the individual strands from 4 of the most highly immunostimulatory duplexes (RS002, RS009, siCyan2 and siRNA9.2). In each case, the U-rich strand elicited greater IFN production from PBMCs (Figure 3), confirming the underlying premise of the analysis and validating the findings of previous studies that TLR 3 is not involved in the sensing of siRNA molecules in isolated human leukocytes (since single-stranded RNA is not a ligand for TLR3).

**Figure 4:**
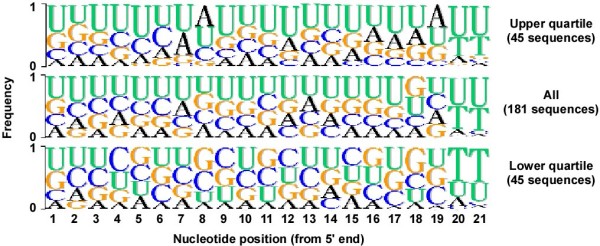
**Logo representation of consensus sequences of TLR7/8 activity**. Alignments of U rich strands from the upper and lower quartiles of replicon inhibition (upper and lower panels respectively), and from the entire set of siRNAs tested (26 duplexes with equal U content in both strands excluded from the analysis).

To identify the sequence determinants of TLR7/8 activation by siRNAs, we performed alignments of the U-rich strands from each of the duplexes. For this analysis, we excluded those siRNAs in which both strands had equal U content, leaving 181 duplexes for analysis. The frequency of each base at each position was determined for the top and bottom quartiles of activity (45 strands each), and this was compared to the relative frequencies of each base in the U-rich strand of the population (Figure 4). From this analysis, several sequence features that promoted or impaired TLR7/8 activation were suggested. Features that appeared to promote TLR7/8 activation were: G bases at positions 2 and 13, A bases at positions 7, 8 and from 16–19, an absence of C bases in positions 16–19, and an absence of G bases from positions 5–8 and 18. Features that appeared to impair TLR7/8 activation were: C bases at positions 4, 9, 10, 12 and 15, and G bases at positions 5, 8, 11 and 16. Most of the duplexes tested included 3' overhangs comprising UU or TT (deoxy base) dinucleotides. We also found that TT overhangs were over represented in the bottom quartile of immunostimulatory activity, suggesting that UU overhangs can, in some cases, contribute to the activation of TLR7/8. These features suggested that the degree of TLR7/8 activation induced by a given duplex is dependent upon stimulatory motifs in the U-rich strand (containing some G and A nucleotides), but is impaired by CG or GC "clamps" which inhibit the release of the active strand from the duplex.

**Figure 5:**
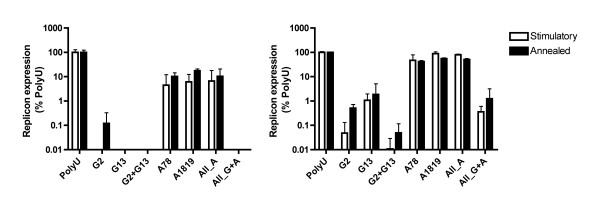
**Innate immune stimulation of PBMCs and pDCs by siRNAs incorporating predicted TLR7/8 stimulatory motifs**. Supernatant from PBMCs (left panel) or pDCs (right panel) treated with 100 nM DOTAP complexed siRNAs (single-stranded U rich strands = Stimulatory; annealed duplexes = Annealed) for 24 hours was applied to Huh7-Luc cells. Luciferase expression was assayed 24 hours after treatment with supernatant. Data is expressed as replicon expression on a log scale to allow visualisation of differences between highly active RNA species. Each point represents the mean of triplicate samples.

**Figure 6:**
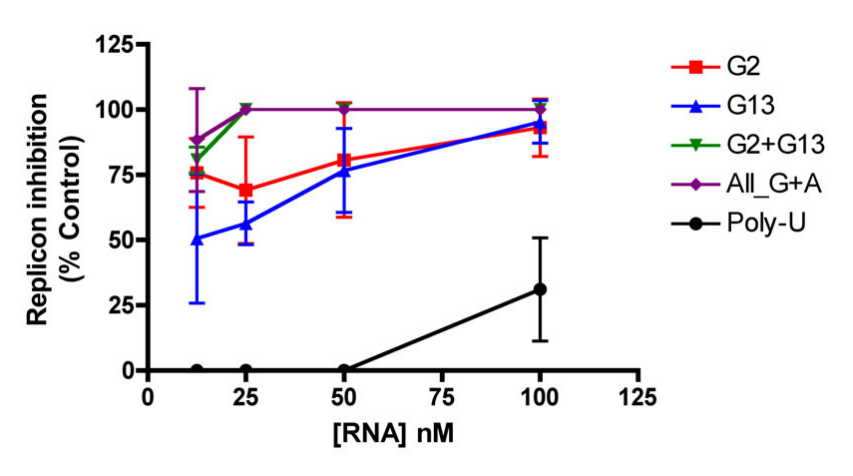
**Innate immune stimulation of PBMCs and pDCs by ssRNAs incorporating predicted TLR7/8 stimulatory motifs**. Supernatant from PBMCs (left panel) or pDCs (right panel) treated with 100 nM DOTAP complexed single-stranded U-rich RNAs for 24 hours was applied to Huh7-Luc cells. Luciferase expression was assayed 24 hours after treatment with supernatant. Each point represents the mean of triplicate samples.

To more specifically investigate the effect of each of the above-mentioned sequence features on the TLR7/8 activation activity of siRNAs, we tested the immunostimulatory activity of a number of siRNAs specifically designed to incorporate these features in a poly-U background. Compared with a poly-U/Poly-A duplex, duplexes containing even a single G-nucleotide at either position 2 or position 13 of the U-rich strand demonstrated strikingly increased immunostimulation of PBMCs (Figure 5). Introduction of A-nucleotides at positions 7 and 8, or 18 and 19 produced more modest increases in activity. Treatment of PBMCs with the U-rich strand alone recapitulated (and in many cases exceeded) the immunostimulatory activity of the duplex. By contrast, none of the corresponding A-rich complementary strands demonstrated activation of TLR7/8 (data not shown). Dose responses for the individual U-rich strands containing G-nucleotides, indicated an additive effect of G-nucleotides at position 2 and 13, but inclusion of A-nucleotides at positions 7, 8, 18 and 19 did not appear to increase activity further (Figure 6). When PBMCs were replaced in the assay by purified pDCs (thus making the assay specific for TLR7), G-nucleotides at positions 2 or 13 again caused substantial increases in immunostimulatory activity, with position 2 appearing to produce a stronger effect than position 13 (Figure 5). In pDCs, however, the inclusion of A-nucleotides in the U-rich strand had only a very modest effect on immunostimulatory activity, and inclusion of A-nucleotides in a sequence containing G-nucleotides at positions 2 and 13 actually decreased immunostimulation. These findings suggest that A nucleotides may preferentially affect recognition of short RNAs by TLR8.

**Figure 7:**
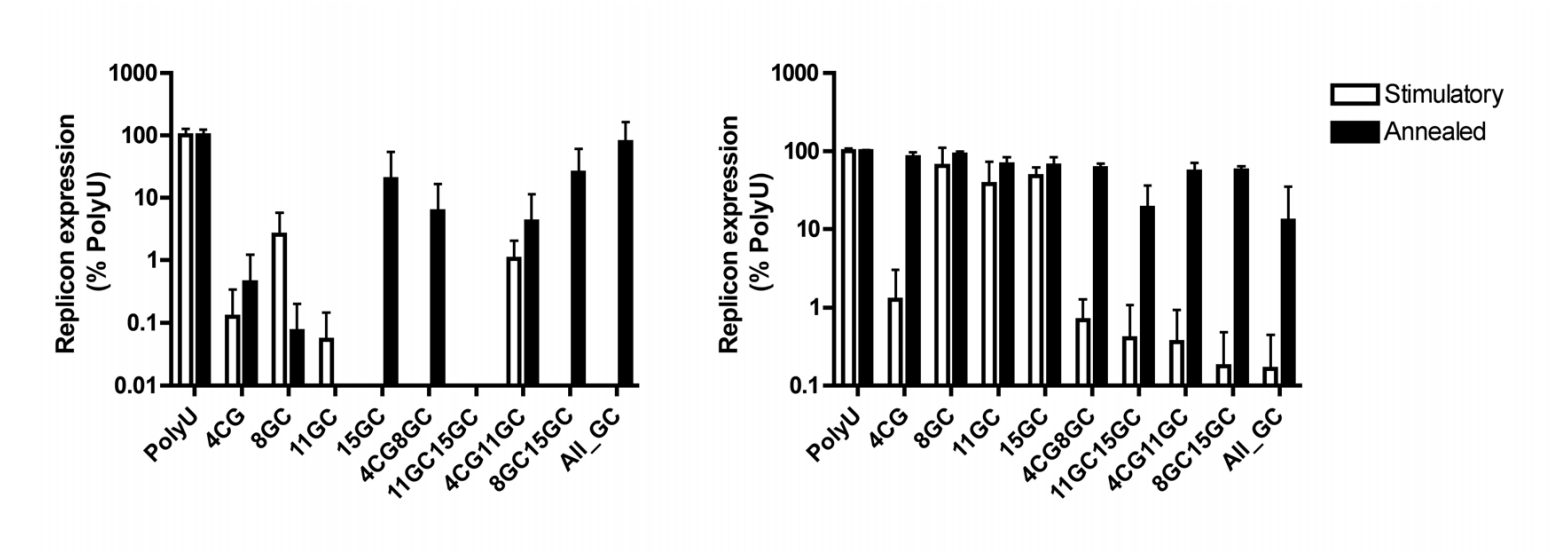
**Innate immune stimulation of PBMCs and pDCs by siRNAs incorporating predicted TLR7/8 inhibitory motifs**. Supernatant from PBMCs (left panel) or pDCs (right panel) treated with 100 nM DOTAP complexed siRNAs (single-stranded U rich strands = Stimulatory; annealed duplexes = Annealed)for 24 hours was applied to Huh7-Luc cells. Luciferase expression was assayed 24 hours after treatment with supernatant. Data is expressed as replicon expression on a log scale to allow visualisation of differences between highly active RNA species. Each point represents the mean of triplicate samples.

Inclusion of features (CG or GC dinucleotides) predicted to decrease the immunostimulatory activity of the duplex generally led to increases in immunostimulation of both PBMCs and pDCs relative to poly-U for both the U-rich single strand and the annealed duplex (Figure 7). However, given the marked enhancement in activity observed for Poly-U oligos containing even a single G-nucleotide (above), it appeared likely that any impairment of TLR7/8 stimulation conferred by increased hybridisation strength may have been masked by the much greater stimulatory activity of the U-rich strand following introduction of a CG or GC "clamp". This hypothesis was supported by the observation that despite the individual U-rich strands of the CG/GC containing duplexes exhibiting potent immunostimulatory activity relative to Poly-U, this activity was largely negated in several of the corresponding duplexes, most notably the RNA duplex (All_GC) which contained 4 CG/GC "clamps". This effect was also observed in purified pDCs, in which all of the CG/GC containing duplexes demonstrated reduced immunostimulatory activity relative to the U-rich strand alone (Figure 7).

**Figure 8:**
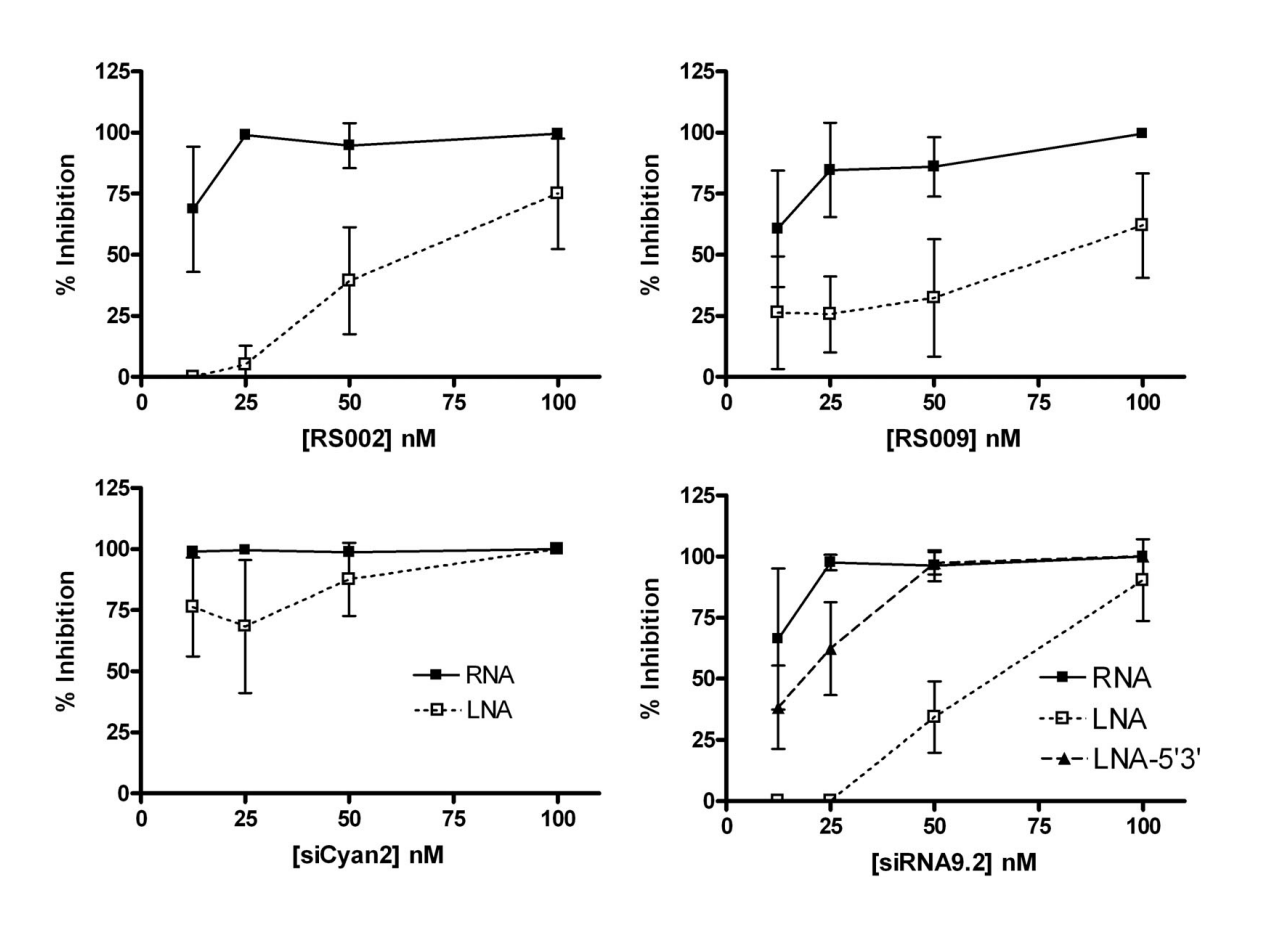
**Innate immune stimulation of PBMCs by LNA modified siRNAs**. Supernatant from PBMCs treated with 100 nM DOTAP complexed siRNAs for 24 hours was applied to Huh7-Luc cells. Luciferase expression was assayed 24 hours after treatment with supernatant. LNA (red) indicates that the non-stimulatory strand contained LNA modifications at positions hybridizing to 4, 5, 8, 9, 11, 12, 15 and 16 of the stimulatory strand. LNA-5'3' (blue) indicates that the non-stimulatory strand contained LNA modifications at positions hybridizing to 1–4, and 16–19 of the stimulatory strand. Each point represents the mean of triplicate samples. Error bars indicate standard deviation.

The above findings suggested that CG or GC clamps interspersed along the length of an siRNA duplex act to inhibit TLR7/8 activation by increasing the strength of hybridization between the two strands, and thus impair the release of stimulatory single-stranded RNA. However, introduction of such clamps into the siRNA duplex complicates the analysis by appearing to increase the stimulatory activity of the active single stranded RNA sequence through the introduction of G nucleotides. To specifically test the effect of increasing the strength of the hybridisation interaction without changing the sequence of the stimulatory strand, we used locked nucleic acid (LNA) chemistry. LNA bases were incorporated into the non-stimulatory strand of 4 highly active duplexes (RS002, RS009, siCyan2 and siRNA9.2) at positions corresponding to 4,5,8,9,11,12,15 and 16 of the stimulatory strand (mimicking the GC clamps tested above), and the immunostimulatory activity of these duplexes was assessed in PBMCs (Figure 8). In each case we found that duplexes containing LNAs were significantly less active than the corresponding fully-ribonucleotide duplex. This finding was in contrast to a previous report, in which an siRNA9.2 duplex including 4 LNA bases at both the 5' and 3' ends of non-stimulatory strand of the duplex was no less immunostimulatory than a fully-ribonucleotide duplex [21]. To assess whether this discrepancy was related to differences in the assay technique, we tested such an LNA modified siRNA in our assay. LNA modification of the 4 terminal hybridizing bases of the non-stimulatory strand did not significantly decrease the immunostimulatory activity of the duplex, a result that was highly consistent with previous findings. The reason that, in this case, LNA modification in the centre of the duplex impairs immunostimulation whereas LNA modification at the termini does not is not clear. It is possible that in terminally modified duplexes the centre section may be sufficiently denatured in the endosome to activate TLR7/8 signalling. Alternatively, it is possible that this specific terminally modified RNA strand forms a hairpin, making it unavailable for duplex formation and thus ineffective in impairing TLR7/8 activation, however, further studies are required to distinguish between these possibilities.

**Figure 9:**
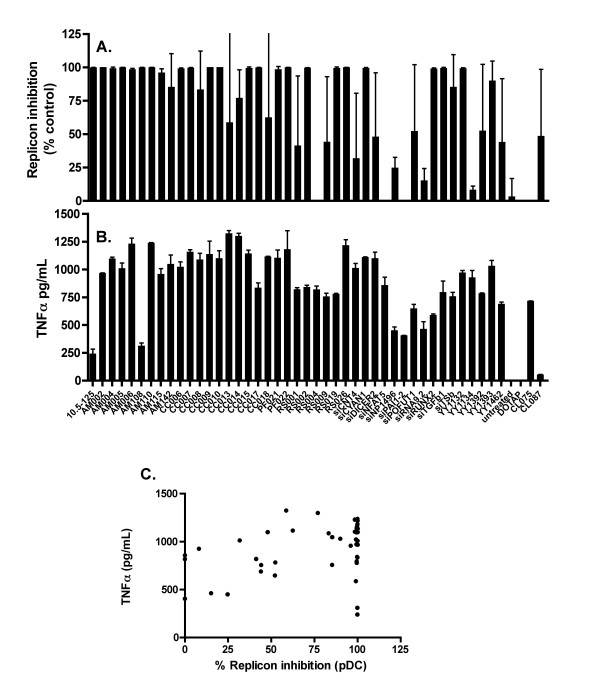
**Innate immune stimulation of pDCs and TNFα production by PBMCs in response to duplex siRNAs**. (A) Supernatant from pDCs treated with 100 nM DOTAP complexed siRNAs for 24 hours was applied to Huh7-Luc cells. Luciferase expression was assayed 24 hours after treatment with supernatant. (B) Supernatant from PBMCs treated with 100 nM DOTAP complexed siRNAs for 24 hours was assayed for TNFα by ELISA. (C) Correlation between data in (A) and (B). Each point represents the mean of triplicate samples. Error bars indicate standard deviation.

It has been proposed that siRNAs activate TLR7 in pDCs leading to IFN production, and/or TLR8 in monocytes leading to IFN and TNFα production [26]. To investigate the sequence specificity of TLR7 and TLR8 activation, we tested 43 of the siRNAs that we had shown to induce IFN production in PBMCs for IFN production in pDCs (TLR7 mediated), and TNFα production in PBMCs (TLR8 mediated). We observed a range of activities for both IFN production from pDCs (further validating the findings of previous studies that TLR 3 is not involved in the sensing of siRNA molecules in isolated human leukocytes, since pDCs do not express TLR3) and TNFα production from PBMCs, although the TLR7 response in pDCs was saturated in many cases (Figure 9A and 9B). The specificity of these assays for TLR7 and TLR8 activation was confirmed by the respective responses to the TLR7 selective agonist CL087, and the TLR8 selective agonist CL075. Given that immunostimulatory siRNAs might be expected to exhibit a preference for stimulation of either TLR7 or TLR8 and that the initial screen for activity (IFN production in PBMCs) measures combined IFN production from TLR7 and TLR8 stimulation, it might be expected that TNFα production from PBMCs and IFN production from pDCs would exhibit negative correlation. However, no such correlation was observed (Figure 9C). For both TNFα production from PBMCs, and IFN production from pDCs, moderate correlation was observed with overall IFN production from PBMCs (data not shown), suggesting that the differences in the affinity of each individual siRNA for its receptor(s) may be masking any trend. However, normalisation of both TNFα production in PBMCs and IFN production in pDC to overall IFN production from PBMCs failed to reveal a negative correlation between IFN production from pDCs and TNFα production (data not shown). Thus, although a small number of individual siRNAs with strong preferences for TLR7 (2 sequences caused more than 50% replicon suppression in pDCs, but secretion of less than 500 pg/mL TNFα in PBMCs or TLR8 (8 of 43 caused less than 50% replicon suppression in pDCs, but secretion of more than 500 pg/mL TNFα in PBMCs) were identified, there were too few such sequences to draw conclusions about sequence determinants of TLR7 or TLR8 preference. For the subset of 43 immunogenic siRNAs described above, we also investigated both IL-8 and IP-10 expression by ELISA. IL-8 expression correlated with TNFα expression (data not shown). IP-10 was induced approximately equally by all sequences tested (data not shown), and so did not allow any discrimination between sequences.

**Figure 10:**
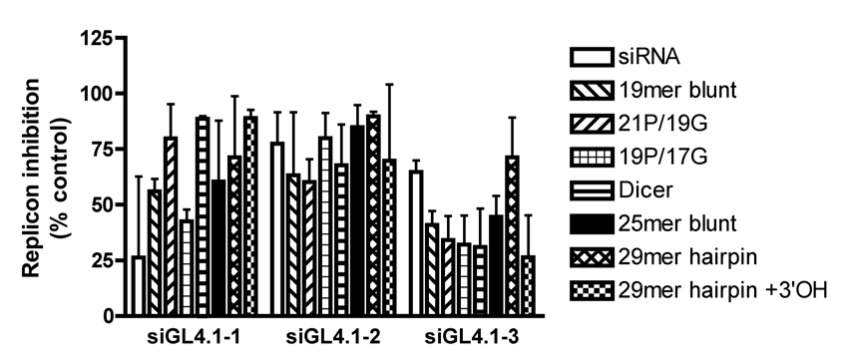
**Immunostimulation of PBMCs by different siRNA formats**. Supernatant from PBMCs treated with 100 nM DOTAP complexed siRNAs (siRNA = standard 19 bp duplex with 2 base 3' overhangs; 19 mer blunt = 19 bp duplex with no overhangs, 21 P/19 G indicates 21 bp passenger strand annealed to a 19 bp guide strand; 19 P/17 G indicates 19 bp passenger strand annealed to a 17 bp guide strand; Dicer = dicer substrate siRNA; 25 mer blunt = 25 bp duplex with no overhangs; 29 mer hairpin = 29 bp hairpin RNA; 29 mer hairpin +3'OH = 29 bp hairpin RNA with 3'-hydroxyl group) for 24 hours was applied to Huh7-Luc cells. Luciferase expression was assayed 24 hours after treatment with supernatant. Each bar represents the mean of triplicate samples. Error bars indicate standard deviation.

The studies described above were all performed using standard format 21 mer siRNAs with 2 base 3' overhangs. However, a number of other siRNA formats have been described in the literature, including siRNAs of varying lengths, "blunt"-ended siRNAs, hairpin RNAs and siRNAs incorporating DNA nucleotides [34-38]. We tested the immunostimulatory properties of a range of different siRNA formats based upon 3 different sequences (see Additional File 1). No discernable trend was detected (Figure 10), although longer sequences (including hairpins), did appear to be generally slightly more immunostimulatory. However, other factor(s) than format (most likely sequence dependent) appear to have been the major determinants of immunogenicity, and given the small number of sequences used (only 3 base sequences) we do not consider these results to be conclusive.

## Conclusion

In the present study we have used a broad-based screening strategy to identify sequence determinants of TLR7/8 activation by standard format duplex siRNA molecules. We found that sequence motifs contained in a single, U-rich strand of the siRNA duplex were a major determinant of activity, as demonstrated by the profound increase in immunostimulation caused by the introduction of even a single G-nucleotide into a poly-U sequence. We also found that the thermodynamic properties of the duplex affected TLR7/8 activation, and that the strength of the hybridisation interaction between the two strands correlated negatively with immunostimulatory activity. Thus, our findings suggest a model of TLR7/8 activation by siRNAs, in which the two strands are denatured in the endosome, and single-stranded, U-rich RNA species activate TLR7/8. In the course of these studies, we have also identified several RNA species that stimulate TLR7/8 more strongly than any previously identified agent that we are aware of. These findings have relevance to the design of siRNAs (particularly for *in vivo *or clinical applications) and to the understanding of the physiological mechanisms of TLR7/8 activation. The highly immunostimulatory RNA species that we have identified may also be useful in the development of pharmacological agents with anti-viral or vaccine adjuvant applications.

## Methods

### Cell culture

Human hepatoma cells (Huh7) containing a stable pFK-I389/NS3-3'/5.1 based, Luciferase expressing HCV replicon (Huh7-Luc) [39] were maintained in Dulbecco's modified Eagle's medium (DMEM, Invitrogen, Carlsbad CA) containing 10% fetal calf serum (FCS, Sigma-Aldrich, St. Louis, MO) and 750 μg/mL G418 (Invitrogen) at 37°C in a 5% CO_2 _incubator.

### PBMC isolation

Buffy coats were obtained from the Australian Red Cross Blood Service. Buffy coat (50 mL) was diluted 1:3 with DM-L wash buffer (PBS + 2% FCS). This mixture was split into 6 separate 25 mL aliquots and each 25 mL of diluted cells layered over 15 mL of RosetteSep DM-L (Stemcell Technologies, Vancouver, Canada) solution. Following centrifugation for 20 min at 1200 *g*, erythrocytes were discarded and the mononuclear band washed with 40 mL DM-L wash buffer. Cells were pooled in 10 mL DM-L wash buffer prior to addition of 25 mL Ammonium Chloride solution (Stem Cell Technologies). Following 5 min incubation on ice the cells were centrifuged at 300 *g *for 5 min and resuspended in 50 mL DM-L wash buffer.

### Plasmacytoid dendritic cell purification

Plasmacytoid dendritic cells (pDCs) were purified by negative selection using a plasmacytoid dendritic cell isolation kit (Miltenyi Biotec, Bergisch Gladbach, Germany) according to the manufacturer's instructions. Briefly, fresh PBMCs were resuspended in MACS buffer (PBS pH 7.2, 0.5% BSA, 2 mM EDTA) and 100 μL PDC biotin antibody cocktail added per 10^8 ^cells. Following incubation on ice for 10 min the cells were washed twice with 10 mL MACS buffer per 10^8 ^cells. Cells were incubated with 100 μL anti-biotin microbeads for 15 min on ice and washed in MACS buffer prior to being loaded on an LS column (Miltenyi Biotec). The unlabelled fraction containing the enriched pDC fraction was collected and cells stained with CD303-FITC (Miltenyi Biotec) and CD45-PerCP (Becton Dickinson, Mountain View, CA). Analysis of pDC purity was performed by flow cytometry using a FACSCalibur flow cytometer (Becton Dickinson).

### Stimulation of TLR signalling in immune cells

The following small molecule TLR ligands were dissolved in DMSO and stored at -20°C until use: Resiquimod (Alexis Biochemicals, Lausen, Switzerland); CL075 and CL087 (both from Invivogen, San Diego, CA). Chloroquine (Sigma-Aldrich, St Louis, MO) and all siRNAs (see Additional File 1) were resuspended in nuclease-free water and stored at -20°C until required. Unless otherwise indicated IFNα-2b (R&D Systems, Minneapolis, MN) was used at a concentration of 100 U/mL. Where indicated PBMCs were treated with up to 10 μM chloroquine and incubated at 37°C in a 5% CO_2 _incubator for 1 h prior to addition of siRNA.

Duplex siRNAs for testing were sourced from 3 different research groups within Johnson & Johnson Research Pty Ltd, and had been designed to a number of targets from different species. RNA oligos and duplex siRNAs were purchased from Sigma-Aldrich).

PBMCs or pDCs (84.5% CD303+, data not shown) were stimulated with either siRNA or known TLR agonists for 24 h prior to supernatant transfer to Huh7-Luc cells, following previously determined experimental protocols [32,33]. Unless otherwise indicated 50,000 PBMCs plated in 96-well plates in 80 μL antibiotic-free 10%FCS-RPMI were stimulated with TLR agonist (final volume of 100 μL/well; maximum 1% DMSO per well) or transfected with siRNA complexed with DOTAP (Roche) (final volume of 100 μL/well; maximum 100 nM siRNA per well). For complexations 10 pmol siRNA was complexed with 0.7 μg DOTAP in 10%FCS-RPMI. The mock transfection control used the highest concentration of DOTAP (0.7 μL DOTAP per well). After addition of stimuli the plates were incubated at 37°C in a 5% CO_2 _incubator for 24 h.

For measurement of IFN production, Huh7-Luc cells were seeded at 7500 cells/well in 80 μL antibiotic-free media (10%FCS-DMEM) in white 96-well plates (Greiner Bio-one, Frickenhausen, Germany). Media was removed 24 h post-seeding and 100 μL PBMCs pre-treated for 24 h with TLR agonist or siRNA was added to each well. After addition of PBMC supernatant the plates were incubated at 37°C in a 5% CO_2 _incubator for 24 h. PBMCs were subsequently removed and proliferation of Huh7 or Huh7-Luc cells determined using the Cell Titre Blue (CTB) assay (Promega, Madison, WI). Following measurement of fluorescence (Fluostar Optima plate reader, BMG Labtech, Offenburg, Germany) the CTB reagent was removed and cells lysed with 30 μL 1× Passive Lysis Buffer (Promega) at room temperature for 10 min. Luminescence was measured every 0.5 s for 5 s using automatic substrate injection (Luciferase assay system, Promega) and average luminescence calculated for each well. TNF-α production was measured by Quantikine ELISA (R&D systems) according to the manufacturer's instructions.

### Bioinformatics and statistical analysis

Statistical analysis was performed using Prism (GraphPad Software, San Diego, CA) and Pipeline Pilot (Accelrys Software, San Diego, CA) software. Logos were generated using Phylo-mLogo 2.3 software [40]. Unless otherwise indicated treatments were performed in triplicate and error bars represent standard deviation.

## Authors' contributions

AG contributed to design and execution of cell assays and contributed to drafting of the manuscript. NN carried out the cell culture assays. AK provided technical experimental assistance as well as assistance in data analysis and presentation. TD performed bioinformatics analysis. MT was involved in siRNA design. GA participated in siRNA design and conception of study. MP performed bioinformatics analysis. LR participated in study design and conception. TP involved in study conception, experimental design and execution, statistical analysis and drafting of manuscript. All authors read and approved the final manuscript.

## Supplementary Material

Additional file 1 **siRNA sequences used for screening**. All siRNA sequences used throughout the manuscript. Click here for file
